# Extraction-depended and thermally-modulated physical and chemical properties of powders produced from cranberry pomace extracts

**DOI:** 10.1016/j.crfs.2023.100664

**Published:** 2023-12-17

**Authors:** Jessica Brzezowska, Aleksandra Hendrysiak, Aneta Wojdyło, Anna Michalska-Ciechanowska

**Affiliations:** Department of Fruit, Vegetable and Plant Nutraceutical Technology, Faculty of Biotechnology and Food Science, Wrocław University of Environmental and Life Sciences, Chełmońskiego 37, 51-630, Wrocław, Poland

**Keywords:** *Vaccinium macrocarpon* L., Pomace valorization, Food sustainability, Phenolic extracts, Inulin, Food safety

## Abstract

Recovering bioactives from botanical by-products in the form of powders has been attempted through a number of multidirectional approaches. Yet understanding the processing of such plant formulations requires dedicated research owing to the manifold factors shaping the quality of powders. Therefore, the study aimed at production of cranberry powders from pomace extracts and to evaluate how different solvent type, carriers and drying techniques modulate their physico-chemical properties. Freeze- and vacuum drying significantly differentiated samples in terms of physical properties, while the extraction solvent and carrier type had substantial impact on chemical ones. For carrier-added products pomace extraction with acidified 50% ethanol resulted in the highest content of identified phenolics in powders (up to 5.87 g · 100 g^−1^ dry matter), while 30% acetone in the lowest (on average, 3.94 g · 100 g^−1^ dry matter). Acetone extraction strengthened the formation of hydroxymethyl-*L*-furfural that was higher when compared to acidified 50% ethanol, while trace amounts were reported for non-acidified counterpart. Similar observation was made in the case of flavan-3-ols. Addition of carriers during powders production led to the lower hydroxymethyl-*L*-furfural formation even down to 74% with regard to carrier-free samples. The study confirmed feasibility of managing cranberry pomace into high-value powders in extraction-depended and thermally-modulated quality matter.

## Highlights

• Cranberry pomace was successfully converted into a phenolic-rich powder.
• Acidified 50% ethanol resulted in the greatest retention of bioactives in products.
• Carrier addition diminished HMF content by 74% compared to carrier-free samples.
• Pomace extraction with 50% ethanol resulted in trace HMF and flavan-3-ols content.
• Vacuum drying ensured the most favorable physical properties of cranberry powders.

## Graphical abstract

**Figure:**
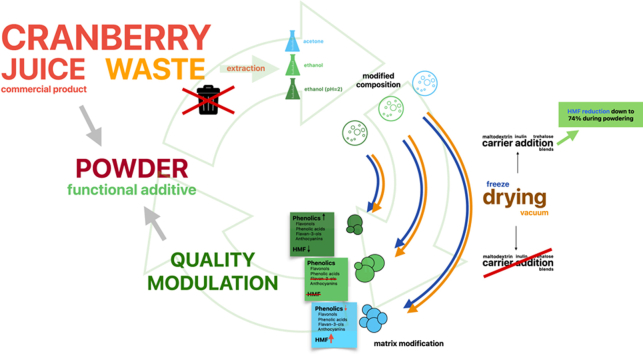

## 1 Introduction

Management of plant-based by-products has become one of the most significant issues linked with EU waste policies, including the European Green Deal (European Commission, 2023; Srivastava and Balakrishnan, 2022). Reutilization of plant production residues is also in accordance with recent movements related to resource-efficient use of biomass, which includes sustainable and circular practices. Furthermore, consumer perception is becoming a key point in preparation of novel food additives based on the botanical-based wastes (Raak et al., 2017). Fruit pomace is an example of inexpensive sources of bioactives that due to its matrix specificity remain unexploited, however progressive research is being undertaken in this direction with usage of fruit by-products as natural food additives and sustainable antimicrobial agent for food packaging.

Nevertheless, the diversity in a chemical composition and structural variations of plant by-products can be a barrier for their reuse by the food and pharmaceutical industries. Usually, botanical wastes were dehydrated by different drying techniques and then extracted by various solvent types (Srivastava and Balakrishnan, 2022). Another solution for their management and thus possible incorporation in the diet or pharmaceuticals is production of powders from phenolics-rich preparations after their *prior* extraction from pomace matrix. Such an approach offers a number of merits over whole pomace powdering, including solubility, increased bioavailability and versatility for industrial use as a food additive, and therefore convenient as an easy-to-handle form. Despite enumerated pros, this technology still poses a challenge as the complex matrix composition of different raw materials requires adjustment of appropriate extraction procedure, i.e., solvent type, ratio, conditions, etc. This step is critically important not only because of the yield of extracted bioactives, but also the profile of other constituents passing into solution during the procedure, which can act as substrates during the formation of potentially harmful process contaminants, or catalyze these reactions evoked under thermal treatment (Michalska-Ciechanowska et al., 2021a, 2021b). A popular solvent used to extract phenolics from plant matrices is acetone (Oszmiański and Krzywicki, 1993). Due to multiple doubts concerning the reasonableness of its use in the context of food safety, the search for other food-compatible solvents has begun. Ethanol, a food-grade solvent, appears to be a reasonable alternative, in terms of food safety and environmentally friendly management of waste generated in the extraction process (Roopchand et al., 2013). The next crucial stage involves converting the liquid form of extracted bioactive into powder, and thus adapting appropriate drying technique along with the process parameters. Freeze-drying, being a low temperature water removal procedure, is commonly recognized as the least impactful, having minimal effect on transformation of the dried matrix. Some studies have shown that the use of high-temperature processing, as vacuum drying, may result in release of individual phenolics from more polymerized structures, thereby enhancing health-promoting properties of powders (Michalska-Ciechanowska et al., 2021a ). However, in some cases, obtaining a powder is only possible after adding a high molecular weight substance, as the product yield may be insufficient or powdering process may not be feasible. Recently, numerous multidirectional studies are focused on exploring the possibility of using carriers with functional properties, such as inulin with prebiotic properties or trehalose, whose structure prevents potential participation in the Maillard reaction (Michalska-Ciechanowska et al., 2021b).

Cranberry, due to its sour and bitter taste, is processed into juice in considerable mer, and pomace generated constitutes the by-product significantly richer in phenolics than the main product itself (Oszmiański et al., 2016). Thus, the research attempted to utilize cranberry pomace, considered a waste, into a high-quality and convenient soluble type powdered product. Subsequently, the study aimed at the comparison of non-GRAS (acetone) and GRAS (ethanol) solvents used for the phenolics extraction from cranberry pomace in order to produce phenolics-concentrated extracts’ powders and track the possible alterations caused either by freeze-drying and vacuum drying as well as different carrier types used for their production. Moreover, bearing in mind the probable formation of process contaminants during processing, the presence of hydroxymethyl-*L*-furfural was monitored.

## 2 Materials and methods

### 2.1 Materials

The fruit (*Vaccinium macrocarpon* L.) obtained from the Rolniczo-Sadownicze Gospodarstwo Doświadczalne ‘Przybroda’ (Rokietnica near Poznań, Poland) was cleaned and crushed with a Thermomix (Wuppertal, Vorkwek, Germany). Pulp was pressed in a laboratory hydraulic press (SRSE, Warsaw, Poland). The pomace was packed in polyethylene bags and stored at −20 °C until extraction. Maltodextrin (dextrose equivalent (DE) 9.3; Przedsiębiorstwo Przemysłu Spożywczego PEPEES S.A., Poland), inulin (Beneo-Orafti, Belgium) and trehalose (Hayashibara Co., Japan) were used as carriers for powders production.

### 2.2 Methods

#### 2.2.1 Extraction and powders obtainment

##### 2.2.1.1 Solvent extraction

Pomace phenolics-rich extracts were prepared according to the patent (Oszmiański and Krzywicki, 1993) and procedure proposed by Roopchand et al. (2013). Defrosted pomace (approx. 15 kg) was mixed with different solvents: 30% acetone, 50% ethanol (*w/w*), and acidified 50% ethanol to pH = 2 (*w/w*) (1:4 *w/v*; experimentally established). The mixture was sonicated for 15 min. After 24 h the solution was sonicated again for 15 min and filtered. Extraction solvent was removed from obtained filtrate using a Unipan 350P vacuum rotary evaporator (Warsaw, Poland). Resulting extract (approx. 6 L for each solvent) was subjected to the column chromatography (Kammerer et al., 2005) using the XAD-16 amberlite polymer resin (Brenntag, Poland) for improved absorption and desorption of phenolics (Gao et al., 2018). After the eluate was collected, eluent was removed by evaporation and remaining aqueous phase consisting of phenolic-rich extract (approx. 500 mL each, 6 °Bx) was frozen (−20 °C) and stored until powders were obtained.

##### 2.2.1.2 Preparation of powders

Cranberry phenolic-rich extracts were divided into seven parts each and mixed with selected carriers (10%; *w/w*): maltodextrin (M), inulin (I), trehalose (T), and their blends, i.e., maltodextrin-inulin (M-I) (1:1; *w/w*), maltodextrin-trehalose (M-T) (1:1; *w/w*), inulin-trehalose (I-T) (1:1; *w/w*). Samples without carrier addition served as controls. Obtained extract-carrier mixes were subjected to drying.

##### 2.2.1.3 Drying

Freeze drying (FD) was performed (*n* = 2) in a FreeZone freeze dryer (Labconco Corp., MO, USA) for 24 h under a vacuum of 65 Pa. The temperature in drying chamber was −60 °C, while heating plates +24 °C. Vacuum drying was done in a Vacucell 111 Eco Line vacuum oven (MMM Medcenter Einrichtungen GmbH, Germany) at a pressure of 0.1 mbar. Samples were dried at 60 °C (VD60) and at 90 °C (VD90), for respectively 22 h and 16 h in two technological replications (*n* = 2). After drying, powders were vacuum packed (Tepro, Poland) and stored at −20 °C until analyzed.

#### 2.2.2 Analytical methods

##### 2.2.2.1 Physical properties

###### 2.2.2.1.1 Moisture content

Moisture content (Mc) was determined in duplicate (*n* = 2) based on method described by Michalska and Lech (2018) and results were expressed in %.

###### 2.2.2.1.2 Water activity

Water activity (*a*_*w*_) was determined by the Dew Point Water Activity Meter 4 TE by Aqua Lab (USA) at 25 °C (*n* = 2).

###### 2.2.2.1.3 Bulk density

Bulk density (*ρ*_*b*_) was performed according to Michalska and Lech (2018) by determining the mass (*m*) to bulk volume (*Vb*) ration, and expressed as g · cm^−3^ (*n* = 2).

###### 2.2.2.1.4 Color

Color was evaluated according to the CIE L*a*b* system using a Minolta Chroma Meter CR-400 colorimeter (Minolta Co. Ltd., Osaka, Japan) (*n* = 3). Based on the measured parameters, C*, H*, ΔE* and browning index (BI) were additionally established using the equations indicated by Azab et al. (2022), which are presented below.

(Equation 1) $$C*=\sqrt{\left(a*\right)2+\left(b*\right)2}$$

(Equation 2) H* = tan^−1^ [b*/a*]

(Equation 3) ΔE* = [(ΔL*)^2^ + (Δa*)^2^ + (Δb*)^2^]^1/2^

(Equation 4) BI = 100/0.17 ((a*+1.75L*)/(5.647L*+a*-3.012b*)-0.31)

##### 2.2.2.2 Chemical properties

###### 2.2.2.2.1 Determination of phenolics and hydroxymethyl-L-furfural by the UPLC method

Approximately 10 mg of each powder (*n* = 2) was extracted using 30% methanol (*v/v*) with ascorbic acid and directed for phenolics analysis by UPLC as described by Michalska-Ciechanowska et al. (2021a) using an Acquity UPLC system (Waters, Milford, USA) with a Q-Tof mass spectrometer (Waters, Manchester, UK). Particular phenolic group identification was done at the subsequent wavelengths: ***λ*** = 280 nm for flavan-3-ols and hydroxymethyl-*L*-furfural, ***λ*** = 320 nm for phenolic acids, ***λ*** = 360 nm for flavonols, and ***λ*** = 520 nm for anthocyanins. Results were elaborated using Empower 3 software and expressed as g or μg · 100 g^−1^ dm (dry matter).

###### 2.2.2.2.2 Determination of antioxidant capacity *in vitro*

The methanol extracts of powders prepared (*n* = 2) according to Michalska et al. (2019) were used for determination of antioxidant capacity measured by ABTS^•+^ radical cation scavenging ability and FRAP assays (Michalska-Ciechanowska et al., 2021a, 2021b) with Synergy H1 spectrophotometer (BioTek Instruments Inc., USA). Results were expressed as mmol Trolox equivalent per 100 g of dm.

#### 2.2.3 Statistical analysis

The results were analyzed using the STATISTICA 13 program (StatSoft, Tulsa, OK, USA). HSD Tukey test was employed to establish significant (P < 0.05) differences between samples. The Principal Component Analysis was done using XLSTAT Software. The relationships amongst particular variables were determined by calculating Pearson's correlation coefficient (*r*), while polar plots were created using the R package ‘ggplot2’.

## 3 Results and discussion

In line with the newest trends in food technology and consumer expectation related to production of modern food merchandises, obtainment of plant-based powders pose a challenge from the technological point of view. A trial of recycling of bioactives from cranberry by-products into valuable foods in order to stabilize phenolics was previously made (Roopchand et al., 2013); however, the evaluation of drying techniques and parameters influence on the phenolics’ alterations during powdering process remained unknown. In the study, the examination of single processing steps, including not only influence of extraction considering the solvent type and specificity of matrix (compositional and structural properties of pomace differing from raw material), but also the impact of liquid extracts conversion into powdered forms was done. Tracking the possible changes of target compounds and formation of hazardous ones present in final products enable to fully characterize quality of powders and to design processing steps that may affect their composition.

### 3.1 Physical properties

#### 3.1.1 Moisture content (Mc)

The Mc of powders ranged from 0.56% to 11.81% and was a resultant of drying technique, carrier addition and type but also the solvent used to obtain cranberry pomace extracts (Table 1).

**Table 1:** The moisture content (%), water activity (*a*_*w*_), bulk density (g · cm^−3^), and color attributes (L*, a*, b*, C*, H*, ΔE* and browning index) of powders produced from cranberry pomace extracts.

Solvent type	Drying technique	Carrier type	Physical parameters									
			Moisture content	Water activity	Bulk density	Color						
						L*	a*	b*	C*	H*	ΔE*	Browning index
30% Acetone	FD	Controls (no carrier)	10.17 ± 0.95 ^c^	0.397 ± 0.039 ^f^	0.06 ± 0.01 ^a^	33.54 ± 0.40 ^f^	31.27 ± 0.24 ^f^	3.23 ± 0.09 ^e^	31.44 ± 0.24 ^e^	5.90 ± 0.17 ^b^	45.97 ± 0.32 ^g^	0.43 ± 0.00 ^e^
	VD60		1.80 ± 0.85 ^a^	0.223 ± 0.004 ^b-d^	0.58 ± 0.10 ^b^	20.84 ± 0.77 ^bc^	8.62 ± 0.12 ^a^	0.71 ± 0.03 ^a^	8.65 ± 0.12 ^a^	4.69 ± 0.22 ^a^	22.56 ± 0.74 ^bc^	0.36 ± 0.00 ^a^
	VD90		2.17 ± 0.02 ^ab^	0.172 ± 0.010 ^ab^	0.72 ± 0.01 ^bc^	19.80 ± 0.57 ^ab^	12.14 ± 0.12 ^b^	1.53 ± 0.05 ^c^	12.24 ± 0.13 ^b^	7.20 ± 0.16 ^c^	23.28 ± 0.43 ^c^	0.39 ± 0.00 ^c^
50% Ethanol	FD		3.44 ± 0.73 ^ab^	0.323 ± 0.017 ^e^	0.15 ± 0.02 ^a^	26.43 ± 0.74 ^d^	21.99 ± 0.51 ^d^	3.49 ± 0.11 ^f^	22.26 ± 0.52 ^d^	9.02 ± 0.06 ^e^	34.56 ± 0.24 ^e^	0.42 ± 0.01 ^e^
	VD60		1.76 ± 0.22 ^a^	0.181 ± 0.007 ^a-c^	0.68 ± 0.04 ^bc^	18.78 ± 0.45 ^a^	8.86 ± 0.56 ^a^	1.25 ± 0.06 ^b^	8.95 ± 0.56 ^a^	8.07 ± 0.50 ^d^	20.80 ± 0.63 ^a^	0.38 ± 0.00 ^b^
	VD90		3.09 ± 0.54 ^ab^	0.136 ± 0.019 ^a^	0.77 ± 0.00 ^c^	19.90 ± 0.13 ^ab^	9.16 ± 0.21 ^a^	0.81 ± 0.02 ^a^	9.20 ± 0.21 ^a^	5.03 ± 0.02 ^a^	21.92 ± 0.06 ^ab^	0.37 ± 0.00 ^ab^
50% Ethanol (pH = 2)	FD		4.99 ± 1.48 ^b^	0.288 ± 0.016 ^de^	0.07 ± 0.00 ^a^	31.14 ± 0.91 ^e^	26.78 ± 0.05 ^e^	3.16 ± 0.10 ^e^	26.97 ± 0.04 ^e^	6.72 ± 0.22 ^c^	41.20 ± 0.71 ^f^	0.42 ± 0.00 ^e^
	VD60		2.76 ± 0.64 ^ab^	0.243 ± 0.001 ^cd^	0.74 ± 0.01 ^bc^	20.28 ± 0.08 ^ab^	14.20 ± 0.03 ^c^	2.08 ± 0.03 ^d^	14.35 ± 0.03 ^c^	8.35 ± 0.12 ^d^	24.84 ± 0.07 ^d^	0.41 ± 0.00 ^d^
	VD90		2.43 ± 0.39 ^ab^	0.153 ± 0.015 ^a^	0.59 ± 0.10 ^bc^	21.57 ± 0.10 ^c^	9.18 ± 0.24 ^a^	0.74 ± 0.02 ^a^	9.21 ± 0.24 ^a^	4.61 ± 0.05 ^a^	23.46 ± 0.18 ^c^	0.36 ± 0.00 ^a^
30% Acetone	FD	M	8.97 ± 0.55 ^de^	0.353 ± 0.028 ^i-k^	0.15 ± 0.00 ^a^	25.20 ± 0.10 ^d^	19.77 ± 0.09 ^h^	1.88 ± 0.01 ^gh^	19.86 ± 0.09 ^h^	5.42 ± 0.01 ^e^	32.09 ± 0.12 ^gh^	0.41 ± 0.00 ^g^
		I	5.76 ± 0.29 ^c^	0.338 ± 0.004 ^h-j^	0.15 ± 0.00 ^a^	32.94 ± 0.43 ^f^	35.14 ± 0.05 ^m^	2.59 ± 0.10 ^j^	35.23 ± 0.04 ^m^	4.22 ± 0.17 ^c^	48.23 ± 0.32 ^l^	0.44 ± 0.00 ^k^
		T	6.98 ± 0.73 ^cd^	0.365 ± 0.014 ^jk^	0.43 ± 0.01 ^b^	21.13 ± 0.52 ^b^	17.50 ± 0.26 ^f^	2.71 ± 0.02 ^j^	17.71 ± 0.25 ^f^	8.79 ± 0.11 ^g^	27.57 ± 0.56 ^e^	0.42 ± 0.00 ^i^
		M-I	9.44 ± 1.50 ^e^	0.393 ± 0.032 ^kl^	0.15 ± 0.01 ^a^	36.96 ± 0.34 ^h^	32.03 ± 0.05 ^k^	1.45 ± 0.06 ^f^	32.07 ± 0.05 ^k^	2.60 ± 0.11 ^a^	48.93 ± 0.26 ^l^	0.41 ± 0.00 ^g^
		M-T	9.68 ± 0.45 ^e^	0.427 ± 0.024 ^l^	0.13 ± 0.01 ^a^	34.17 ± 0.07 ^g^	30.67 ± 0.20 ^j^	2.09 ± 0.02 ^i^	30.74 ± 0.21 ^j^	3.90 ± 0.02 ^c^	45.97 ± 0.11 ^k^	0.42 ± 0.00 ^h^
		I-T	10.47 ± 1.30 ^e^	0.481 ± 0.007 ^m^	0.18 ± 0.00 ^a^	33.27 ± 0.42 ^f^	32.73 ± 0.36 ^l^	2.99 ± 0.13	32.86 ± 0.37 ^l^	5.22 ± 0.18 ^de^	46.76 ± 0.04 ^k^	0.43 ± 0.00 ^j^
	VD60	M	3.28 ± 0.11 ^b^	0.206 ± 0.003 ^c-f^	0.71 ± 0.03 ^c-e^	19.47 ± 0.07 ^a^	5.87 ± 0.04 ^a^	−0.41 ± 0.03 ^b^	5.88 ± 0.04 ^a^	356.00 ± 0.32 ^h^	20.34 ± 0.06 ^a^	0.34 ± 0.00 ^a^
		I	3.26 ± 0.14 ^b^	0.296 ± 0.001 ^gh^	0.63 ± 0.02 ^cd^	25.29 ± 0.19 ^d^	19.80 ± 0.21 ^h^	1.04 ± 0.04 ^de^	19.83 ± 0.20 ^h^	3.00 ± 0.14 ^ab^	32.13 ± 0.26 ^g^^-^^i^	0.40 ± 0.00 ^f^
		T	3.03 ± 0.29 ^ab^	0.307 ± 0.007 ^g-i^	0.70 ± 0.02 ^c-e^	27.18 ± 0.29 ^e^	21.14 ± 0.20 ^i^	1.92 ± 0.02 ^hi^	21.23 ± 0.20 ^i^	5.20 ± 0.09 ^de^	34.48 ± 0.35 ^j^	0.41 ± 0.00 ^g^
		M-I	3.12 ± 0.05 ^ab^	0.226 ± 0.004 ^ef^	0.74 ± 0.00 ^de^	23.30 ± 0.14 ^c^	10.62 ± 0.10 ^b^	−0.10 ± 0.00 ^c^	10.62 ± 0.10 ^b^	359.46 ± 0.01 ^j^	25.60 ± 0.17 ^c^	0.36 ± 0.00 ^b^
		M-T	2.89 ± 0.44 ^ab^	0.257 ± 0.002 ^fg^	0.68 ± 0.00 ^c-e^	20.99 ± 0.10 ^b^	10.45 ± 0.13 ^b^	1.11 ± 0.01 ^e^	10.51 ± 0.13 ^b^	6.07 ± 0.13 ^f^	23.48 ± 0.14 ^b^	0.38 ± 0.00 ^c^
		I-T	2.84 ± 0.08 ^ab^	0.235 ± 0.005 ^ef^	0.74 ± 0.04 ^de^	25.41 ± 0.32 ^d^	18.57 ± 0.12 ^g^	1.74 ± 0.01 ^g^	18.65 ± 0.12 ^g^	5.36 ± 0.03 ^e^	31.52 ± 0.33 ^g^	0.40 ± 0.00 ^f^
	VD90	M	1.37 ± 0.29 ^ab^	0.097 ± 0.011 ^a^	0.64 ± 0.01 ^cd^	21.16 ± 0.04 ^b^	13.75 ± 0.04 ^d^	1.14 ± 0.03 ^e^	13.80 ± 0.04 ^d^	4.74 ± 0.10 ^d^	25.26 ± 0.03 ^c^	0.39 ± 0.00 ^de^
		I	1.03 ± 0.12 ^ab^	0.107 ± 0.014 ^ab^	0.84 ± 0.11 ^e^	23.54 ± 0.08 ^c^	12.30 ± 0.09 ^c^	−0.66 ± 0.02 ^a^	12.31 ± 0.08 ^c^	356.91 ± 0.09 ^i^	26.57 ± 0.11 ^d^	0.36 ± 0.00 ^b^
		T	1.09 ± 0.19 ^ab^	0.152 ± 0.008 ^bc^	0.45 ± 0.11 ^b^	27.53 ± 0.56 ^e^	18.10 ± 0.16 ^fg^	1.02 ± 0.09 ^de^	18.13 ± 0.17 ^fg^	3.22 ± 0.25 ^b^	32.96 ± 0.40 ^i^	0.39 ± 0.00 ^d^
		M-I	1.33 ± 0.26 ^ab^	0.192 ± 0.007 ^c-e^	0.72 ± 0.01 ^de^	23.73 ± 0.13 ^c^	16.79 ± 0.56 ^e^	0.93 ± 0.10 ^d^	16.81 ± 0.56 ^e^	3.15 ± 0.25 ^b^	29.08 ± 0.30 ^f^	0.39 ± 0.00 ^e^
		M-T	0.99 ± 0.18 ^a^	0.172 ± 0.009 ^cd^	0.80 ± 0.01 ^de^	26.95 ± 0.32 ^e^	18.45 ± 0.36 ^g^	1.09 ± 0.03 ^de^	18.48 ± 0.36 ^g^	3.37 ± 0.04 ^b^	32.68 ± 0.46 ^hi^	0.39 ± 0.00 ^de^
		I-T	2.83 ± 0.24 ^ab^	0.215 ± 0.001 ^d-f^	0.56 ± 0.00 ^bc^	23.50 ± 0.11 ^c^	10.96 ± 0.21 ^b^	−0.15 ± 0.06 ^c^	10.96 ± 0.21 ^b^	359.20 ± 0.32 ^j^	25.93 ± 0.14 ^cd^	0.36 ± 0.00 ^b^
50% Ethanol	FD	M	6.98 ± 1.39 ^d-f^	0.284 ± 0.005 ^f^	0.12 ± 0.00 ^a^	23.67 ± 0.08 ^gh^	21.65 ± 0.23 ^g^	1.84 ± 0.02 ^i^	21.73 ± 0.23 ^g^	4.86 ± 0.05 ^de^	32.13 ± 0.10 ^e^^-^^g^	0.42 ± 0.00 ^e^^-^^g^
		I	2.73 ± 0.38 ^a-c^	0.282 ± 0.014 ^f^	0.14 ± 0.00 ^ab^	36.08 ± 0.07 ^k^	35.28 ± 0.02 ^j^	−0.43 ± 0.02 ^b-d^	35.29 ± 0.02 ^j^	359.31 ± 0.04 ^j^	50.47 ± 0.06 ^j^	0.41 ± 0.00 ^e^
		T	4.99 ± 1.39 ^c-e^	0.402 ± 0.014 ^h^	0.18 ± 0.00 ^ab^	27.06 ± 0.73 ^ij^	28.76 ± 0.25 ^i^	3.22 ± 0.22 ^l^	28.94 ± 0.27 ^i^	6.39 ± 0.37 ^f^	39.63 ± 0.30 ^i^	0.44 ± 0.01 ^i^
		M-I	9.75 ± 0.22 ^f^	0.346 ± 0.017 ^g^	0.15 ± 0.00 ^ab^	28.33 ± 0.74 ^j^	28.38 ± 0.05 ^i^	2.35 ± 0.17 ^j^	28.48 ± 0.06 ^i^	4.73 ± 0.34 ^de^	40.17 ± 0.48 ^i^	0.43 ± 0.00 ^f^^-^^h^
		M-T	8.07 ± 1.31 ^ef^	0.221 ± 0.014 ^de^	0.15 ± 0.00 ^ab^	26.26 ± 0.21 ^ij^	23.24 ± 0.04 ^h^	1.44 ± 0.02 ^h^	23.29 ± 0.04 ^h^	3.55 ± 0.06 ^bc^	35.10 ± 0.17 ^h^	0.41 ± 0.00 ^e^
		I-T	6.70 ± 0.47 ^d-f^	0.290 ± 0.015 ^f^	0.18 ± 0.01 ^ab^	27.93 ± 0.25 ^j^	28.03 ± 0.11 ^i^	2.76 ± 0.09 ^k^	28.16 ± 0.10 ^i^	5.62 ± 0.18 ^ef^	39.66 ± 0.23 ^i^	0.43 ± 0.00 ^g^^-^^i^
	VD60	M	1.01 ± 0.13 ^a^	0.099 ± 0.003 ^a^	0.78 ± 0.05 ^de^	17.56 ± 0.54 ^bc^	9.21 ± 1.29 ^a^	0.50 ± 0.19 ^f^	9.22 ± 1.29 ^a^	3.01 ± 0.78 ^b^	19.85 ± 1.07 ^ab^	0.37 ± 0.01 ^bc^
		I	0.92 ± 0.08 ^a^	0.090 ± 0.005 ^a^	0.73 ± 0.03 ^de^	18.80 ± 0.52 ^cd^	11.23 ± 0.25 ^b^	−0.56 ± 0.09 ^ab^	11.24 ± 0.26 ^b^	357.15 ± 0.38 ^i^	21.91 ± 0.58 ^c^	0.37 ± 0.00 ^b^
		T	3.29 ± 1.01 ^a-c^	0.229 ± 0.000 ^de^	0.56 ± 0.03 ^cd^	22.44 ± 0.57 ^fg^	20.30 ± 0.06 ^f^	1.27 ± 0.06 ^gh^	20.34 ± 0.06 ^f^	3.59 ± 0.16 ^bc^	30.29 ± 0.38 ^e^	0.42 ± 0.00 ^e^
		M-I	1.88 ± 0.40 ^a-c^	0.118 ± 0.004 ^ab^	0.70 ± 0.02 ^de^	16.11 ± 1.67 ^ab^	9.56 ± 0.37 ^a^	−0.14 ± 0.06 ^de^	9.56 ± 0.37 ^a^	359.14 ± 0.36 ^j^	18.75 ± 1.54 ^a^	0.37 ± 0.01 ^bc^
		M-T	1.36 ± 0.32 ^ab^	0.155 ± 0.002 ^bc^	0.55 ± 0.10 ^cd^	14.22 ± 0.88 ^a^	12.93 ± 0.18 ^c^	1.00 ± 0.05 ^g^	12.97 ± 0.18 ^c^	4.44 ± 0.17 ^cd^	19.26 ± 0.53 ^a^	0.42 ± 0.01 ^ef^
		I-T	3.70 ± 0.32 ^a-d^	0.263 ± 0.000 ^ef^	0.85 ± 0.17 ^de^	17.87 ± 1.08 ^bc^	19.79 ± 0.23 ^ef^	1.48 ± 0.02 ^h^	19.84 ± 0.23 ^ef^	4.28 ± 0.04 ^cd^	26.71 ± 0.84 ^d^	0.44 ± 0.01 ^hi^
	VD90	M	1.48 ± 0.10 ^ab^	0.075 ± 0.009 ^a^	0.57 ± 0.05 ^cd^	19.73 ± 0.02 ^c-e^	8.42 ± 0.08 ^a^	−0.44 ± 0.08 ^bc^	8.43 ± 0.08 ^a^	357.01 ± 0.53 ^hi^	21.46 ± 0.03 ^bc^	0.35 ± 0.00 ^a^
		I	1.77 ± 0.47 ^a-c^	0.075 ± 0.011 ^a^	0.79 ± 0.00 ^de^	25.32 ± 0.12 ^hi^	18.93 ± 1.04 ^de^	−0.23 ± 0.06 ^c-e^	18.93 ± 1.04 ^de^	359.31 ± 0.22 ^j^	31.62 ± 0.72 ^ef^	0.39 ± 0.00 ^d^
		T	1.83 ± 0.96 ^a-c^	0.161 ± 0.018 ^bc^	0.39 ± 0.14 ^bc^	27.26 ± 1.49 ^ij^	20.05 ± 0.24 ^ef^	−0.10 ± 0.12 ^e^	20.05 ± 0.24 ^ef^	359.56 ± 0.16 ^j^	33.84 ± 1.25 ^gh^	0.39 ± 0.00 ^d^
		M-I	0.56 ± 0.52 ^a^	0.093 ± 0.012 ^a^	0.69 ± 0.07 ^de^	20.99 ± 0.03 ^d-f^	9.17 ± 0.21 ^a^	−0.81 ± 0.04 ^a^	9.20 ± 0.21 ^a^	354.97 ± 0.35 ^g^	22.92 ± 0.06 ^c^	0.35 ± 0.00 ^a^
		M-T	1.65 ± 0.71 ^ab^	0.105 ± 0.012 ^a^	0.60 ± 0.01 ^c-e^	21.53 ± 0.36 ^e-g^	8.88 ± 0.20 ^a^	−0.61 ± 0.05 ^ab^	8.90 ± 0.20 ^a^	356.07 ± 0.26 ^h^	23.29 ± 0.41 ^c^	0.35 ± 0.00 ^a^
		I-T	4.47 ± 1.73 ^b-d^	0.187 ± 0.016 ^cd^	0.79 ± 0.03 ^de^	27.20 ± 0.20 ^ij^	18.29 ± 0.04 ^d^	0.37 ± 0.04 ^f^	18.29 ± 0.04 ^d^	1.16 ± 0.12 ^a^	32.78 ± 0.14 ^fg^	0.39 ± 0.00 ^cd^
50% Ethanol (pH = 2)	FD	M	6.24 ± 1.72 ^de^	0.346 ± 0.031 ^f^	0.11 ± 0.01 ^a^	29.71 ± 0.13 ^e^	28.26 ± 0.38 ^f^	2.20 ± 0.10 ^f^	28.35 ± 0.39 ^f^	4.45 ± 0.14 ^d^^-^^f^	41.06 ± 0.20 ^i^	0.42 ± 0.00 ^fg^
		I	5.96 ± 1.32 ^c-e^	0.276 ± 0.007 ^e^	0.17 ± 0.00 ^ab^	33.77 ± 0.16 ^g^	36.77 ± 0.03 ^i^	1.99 ± 0.08 ^f^	36.82 ± 0.03 ^i^	3.10 ± 0.12 ^c^	49.96 ± 0.08 ^k^	0.43 ± 0.00 ^gh^
		T	7.05 ± 0.74 ^e^	0.375 ± 0.010 ^f^	0.29 ± 0.01 ^b^	26.51 ± 0.60 ^d^	30.27 ± 0.26 ^g^	4.18 ± 0.20 ^i^	30.56 ± 0.29 ^g^	7.85 ± 0.31 ^h^	40.46 ± 0.18 ^i^	0.46 ± 0.01 ^j^
		M-I	11.81 ± 1.07 ^f^	0.394 ± 0.007 ^f^	0.18 ± 0.00 ^ab^	31.40 ± 1.15 ^f^	34.24 ± 0.14 ^h^	2.71 ± 0.29 ^g^	34.35 ± 0.16 ^h^	4.52 ± 0.46 ^ef^	46.54 ± 0.66 ^j^	0.44 ± 0.01 ^hi^
		M-T	3.29 ± 0.40 ^a-d^	0.364 ± 0.015 ^f^	0.14 ± 0.00 ^ab^	33.02 ± 0.28 ^g^	31.48 ± 0.04 ^g^	2.16 ± 0.06 ^f^	31.55 ± 0.05 ^g^	3.93 ± 0.11 ^de^	45.68 ± 0.20 ^j^	0.42 ± 0.00 ^fg^
		I-T	4.97 ± 1.29 ^b-e^	0.372 ± 0.012 ^f^	0.24 ± 0.00 ^ab^	31.27 ± 0.36 ^f^	34.52 ± 0.41 ^h^	3.56 ± 0.04 ^h^	34.70 ± 0.41 ^h^	5.89 ± 0.05 ^g^	46.71 ± 0.09 ^j^	0.45 ± 0.00 ^i^
	VD60	M	2.54 ± 0.17 ^ab^	0.187 ± 0.006 ^c^	0.75 ± 0.06 ^ef^	18.08 ± 0.29 ^a^	10.56 ± 0.36 ^b^	0.55 ± 0.03 ^c^	10.57 ± 0.36 ^b^	3.00 ± 0.06 ^c^	20.95 ± 0.33 ^a^	0.38 ± 0.00 ^bc^
		I	1.54 ± 0.00 ^a^	0.216 ± 0.003 ^cd^	0.74 ± 0.01 ^d-f^	21.53 ± 0.21 ^b^	16.14 ± 0.43 ^d^	0.12 ± 0.03 ^b^	16.14 ± 0.43 ^d^	0.44 ± 0.10 ^a^	26.91 ± 0.13 ^ef^	0.39 ± 0.00 ^cd^
		T	2.10 ± 0.46 ^ab^	0.253 ± 0.001 ^de^	0.63 ± 0.01 ^c-f^	21.71 ± 0.21 ^b^	15.12 ± 0.14 ^d^	1.01 ± 0.05 ^d^	15.16 ± 0.14 ^d^	3.83 ± 0.14 ^d^	26.48 ± 0.25 ^de^	0.39 ± 0.00 ^d^
		M-I	2.86 ± 0.43 ^a-d^	0.238 ± 0.002 ^c-d^	0.62 ± 0.12 ^c-e^	18.72 ± 0.77 ^a^	13.34 ± 1.74 ^c^	0.53 ± 0.14 ^c^	13.35 ± 1.74 ^c^	2.25 ± 0.34 ^b^	23.03 ± 1.11 ^b^	0.39 ± 0.01 ^d^
		M-T	3.11 ± 0.37 ^a-d^	0.285 ± 0.002 ^e^	0.69 ± 0.06 ^d-f^	19.43 ± 0.81 ^a^	15.89 ± 0.45 ^d^	1.28 ± 0.14 ^de^	15.94 ± 0.45 ^d^	4.59 ± 0.39 ^f^	25.14 ± 0.36 ^c^	0.41 ± 0.01 ^e^
		I-T	2.30 ± 0.00 ^ab^	0.282 ± 0.000 ^e^	0.66 ± 0.00 ^d-f^	21.04 ± 0.77 ^b^	18.93 ± 0.58 ^e^	1.34 ± 0.05 ^e^	18.98 ± 0.58 ^e^	4.05 ± 0.11 ^d^^-^^f^	28.33 ± 0.92 ^g^	0.42 ± 0.00 ^ef^
	VD90	M	4.15 ± 1.89 ^a-e^	0.124 ± 0.016 ^ab^	0.63 ± 0.01 ^d-f^	21.81 ± 0.16 ^b^	7.55 ± 0.09 ^a^	−0.52 ± 0.03 ^a^	7.57 ± 0.09 ^a^	356.06 ± 0.31 ^k^	23.09 ± 0.16 ^b^	0.35 ± 0.00 ^a^
		I	2.58 ± 0.51 ^a-c^	0.124 ± 0.017 ^ab^	0.76 ± 0.02 ^ef^	23.46 ± 0.04 ^c^	10.08 ± 0.08 ^b^	−0.83 ± 0.03 ^a^	10.11 ± 0.08 ^b^	355.31 ± 0.22 ^j^	25.55 ± 0.05 ^cd^	0.35 ± 0.00 ^a^
		T	3.00 ± 0.40 ^a-d^	0.180 ± 0.026 ^bc^	0.46 ± 0.07 ^c^	24.77 ± 0.07 ^c^	13.39 ± 0.06 ^c^	0.17 ± 0.01 ^b^	13.39 ± 0.06 ^c^	0.71 ± 0.02 ^a^	28.16 ± 0.08 ^fg^	0.37 ± 0.00 ^b^
		M-I	2.23 ± 0.28 ^ab^	0.079 ± 0.011 ^a^	0.79 ± 0.02 ^f^	21.70 ± 0.05 ^b^	7.71 ± 0.11 ^a^	−0.78 ± 0.01 ^a^	7.75 ± 0.11 ^a^	354.20 ± 0.12 ^i^	23.05 ± 0.08 ^b^	0.34 ± 0.00 ^a^
		M-T	1.33 ± 0.29 ^a^	0.188 ± 0.021 ^c^	0.57 ± 0.05 ^cd^	21.90 ± 0.06 ^b^	7.80 ± 0.14 ^a^	−0.54 ± 0.02 ^a^	7.82 ± 0.14 ^a^	356.04 ± 0.07 ^k^	23.25 ± 0.10 ^b^	0.35 ± 0.00 ^a^
		I-T	3.31 ± 0.11 ^a-d^	0.234 ± 0.022 ^c-e^	0.75 ± 0.05 ^ef^	26.37 ± 0.10 ^d^	16.36 ± 0.05 ^d^	0.61 ± 0.01 ^c^	16.37 ± 0.05 ^d^	2.15 ± 0.02 ^b^	31.03 ± 0.11 ^h^	0.38 ± 0.00 ^b^^-^^d^

FD – freeze-drying; VD60 – vacuum drying at 60 °C; VD90 – vacuum drying at 90 °C; M – maltodextrin; I – inulin; T – trehalose; M-I – blend composed of maltodextrin and inulin; M-T – blend composed of maltodextrin and trehalose; I-T – blend composed of inulin and trehalose; ^a, b, c, …^– different letters within groups: controls, carrier-added samples (30% acetone), carrier-added samples (50% ethanol), carrier-added samples (50% acidified ethanol) standing for different cranberry pomace extract powders indicate significant differences (ANOVA, HSD Tukey, P < 0.05).

Among samples analyzed, FD led to products with the highest Mc regardless of solvent used for pomace extraction, while VD60 and VD90 resulted in comparable values. The exceptions were control samples after 50% ethanol extraction, for which FD and VD90 yielded in products with relatively similar Mc. This was contrary to expectations, as generally higher temperatures during drying reduce moisture content of powders, however in this case the interplay between matrix composition and applied processing generated ambiguous observations. One possible explanation could be the faster crust formation in matrix during drying, which might hinder evaporation of water and leave it trapped in the product as observed for red pepper powder (Çalışkan Koç, 2020). In the case of freeze-dried powders, those obtained by application of acetone showed the highest moisture content values among the solvents used, both for the control (10.17%) and the products with carriers (8.55%, averaged value). The inclusion of inulin yielded powders with the lowest average Mc values for phenolic-rich samples extracted with acetone and 50% ethanol, while the I-T blend gave the highest, regardless of drying technique. A different pattern was observed for products obtained from acidified 50% ethanol extracts, for which the M-I mixture resulted in the highest and M-T in the lowest mean values of this parameter. These differences might be connected to various carriers’ stability in a given matrix composition and under specific processing conditions. For instance, ambiguous inulin response was observed when subjected to changing thermal and pH conditions (Li et al., 2019; Ozyurt and Ötles, 2016), which, as a result, can lead to divergent properties of final product, including moisture content.

#### 3.1.2 Water activity (a_w_)

Water activity ranged between 0.075 and 0.481 (Table 1). FD resulted in the highest *a*_*w*_, while VD90 in the lowest, for both control powders and those with carriers (averaged values). When solvent type was considered, acetone yielded powders with the highest water activity, while 50% ethanol and its acidified counterpart gave comparable results. This may be related to the extraction of different compounds from cranberry pomace, including various chemical structures or in diverse quantities and therefore with different water-binding abilities (Jin et al., 2019). Taking into account carrier type, inulin proved to be the best for ethanol-extracted samples toward low *a*_*w*_, while maltodextrin for those obtained with acetone application. The I-T mixture caused the highest *a*_*w*_ values exerted by powders obtained after acetone and acidified 50% ethanol extracted samples, while trehalose (followed by I-T blend) in case of those produced with 50% ethanol application, regardless of the drying technique. Overall, powders may be considered as microbiologically stable, as the *a*_*w*_ did not exceed 0.6 value (Fontana, 2020).

#### 3.1.3 Bulk density (ρ_b_)

The *ρ*_*b*_ of cranberry pomace extract powders ranged from 0.06 to 0.85 g · cm^−3^ (Table 1). The drying techniques the most differentiated samples in terms of *ρ*_*b*_ since the lowest bulk density was reported for FD powders, while VD60 and VD90 resulted in products with comparable values, for control and carrier-added samples (Table 1). The differences may be due to structural particularities caused by different drying parameters. Moreover, strong negative correlations were found between bulk density and moisture content (*r* = −0.72) as well as water activity (*r* = −0.71). When carriers were considered, for freeze-dried samples trehalose and I-T blend gave powders with higher bulk density. On the other hand, for vacuum-dried samples at 90 °C, the same single carrier resulted in powders with the lowest values. Overall, as the high bulk density is desirable (savings in packaging and shipping costs), the vacuum-dried powders seem to be the most attractive products from economical and practical point of view (Barbosa-Cánovas et al., 2005).

#### 3.1.4 Color

In almost every case, freeze-drying yielded powders with higher values of L*, a* and b* coordinates, compared to VD60 and VD90 controls and carrier-added samples (Table 1). Acetone and acidified 50% ethanol resulted in products lighter and more yellow than those extracted with 50% ethanol, with exception of controls in case of b* parameter (comparable results). Regardless solvent type and drying technique, inulin led to powders obtainment with the highest L* and a* coordinates, while maltodextrin the lowest. Moreover, trehalose and its blends gave products with the highest b* parameter values. Nevertheless, the discrepancies between values were within a very narrow range, and thus the differences were visually imperceptible. When considering chroma parameter (C*) which stands for color purity and intensity, the highest values were noted for freeze-dried samples, regardless of the carrier addition or solvent used for pomace extraction, while vacuum drying yielded comparable powders in this regard. Interestingly, maltodextrin turned out to lower this parameter compared to other carriers when lyophilization was applied for powders production. In terms of the hue angle (H*), which describes the color perception, all powders were found to be in the range 10° – 0° – 350°, indicating their intense red color. Although the differences between the values analyzed were statistically significant, due to the narrow range of hue angle variation between the samples (Table 1), distinctions in the perception of the color of the powders may be barely noticeable without the use of analytical tools. However, when analyzing the results of the ΔE* parameter, which expresses the total color change, it was found that all powders showed discernible (1.5 < ΔE* < 5.0) and even evident (ΔE* > 5.0) differences in color change to the human eye (Lacerda et al., 2016). Moreover, similarly to the C* coordinate, ΔE* was found to be highest for freeze-dried samples and comparable for vacuum-dried samples at 60 and 90 °C, but in this case the addition of trehalose seemed to alleviate the differences between the drying techniques used. The type of solvent used for the extraction of compounds from cranberry pomace did not considerably affect these parameters. Finally, in order to follow the process-induced changes related to the formation of new compounds responsible for the color change, the browning index was calculated, which indicates the brown color purity and can also be an indicator of the alterations that usually (but not exclusively) occur under thermal treatment and storage (Pathare et al., 2013). In this study, the BI ranged between 0.34 up to 0.46 (Table 1) and therefore, it can be concluded that the powders obtained were highly resistant in this respect and that the technological treatment applied (type of solvent, addition and type of carrier, as well as the drying method) did not influence the matrix undergoing processing substantially.

### 3.2 Chemical properties

#### 3.2.1 Extraction

Previously, attempts were made to extract phenolics from fruit by-products and various solvents as well as additives were tested (Oszmiański and Krzywicki, 1993; White et al., 2010b) with the emphasis on the search for an effective food-compatible solvent. Water with different pH range (2–5) and 50% ethanol solutions were used for extraction of phenolics from fruit pomace with water being significantly less effective (Roopchand et al., 2013). Thus, in the study the 30% acetone (commonly used solvent), 50% ethanol and acidified 50% ethanol (as more food-compatible solvents) were compared for extraction of these constituents.

In order to track changes caused by drying, cranberry pomace extracts were produced by removing ballast substances to examine the alterations of major phenolics group without influence of other cranberry pomace components. An absorber technology was used due to high selectivity, absorption capacity, economic feasibility and low toxicity when compared to other separation techniques (Soto et al., 2011), excluding toxic solvents that could cause degradation of selected bioactives. Additionally, previous study on cranberry products elucidated that matrix composition (juice, sugar-free juice extract) plays an important role and significantly influences presence and content of particular phenolics in resulting powders, and therefore their quality (Michalska et al., 2018), which should be assessed in light of these differences.

#### 3.2.2 Phenolics determination

##### 3.2.2.1 Controls (no carrier addition)

**Fig. 1:**
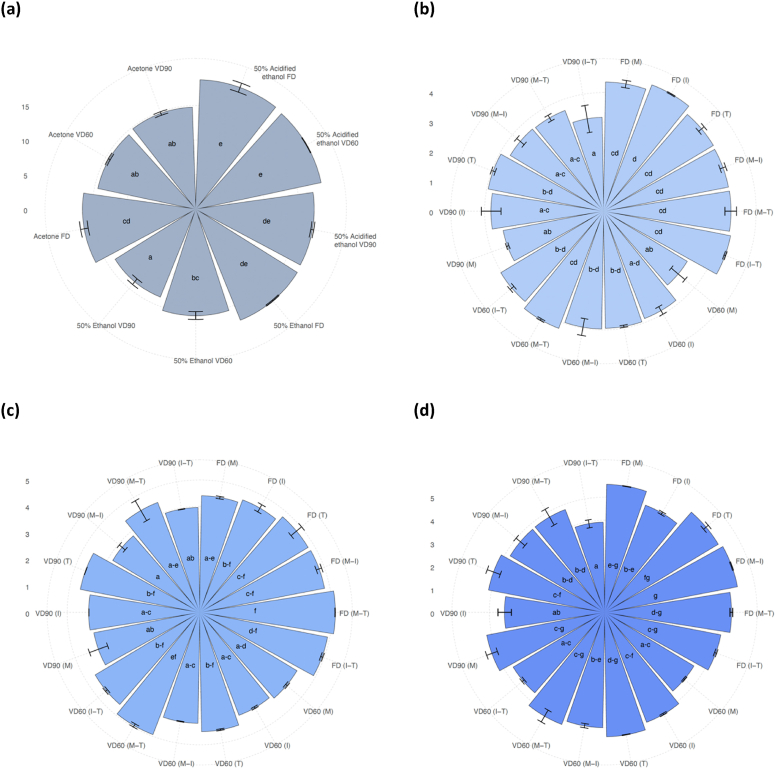
The sum of phenolics [g · 100 g^−1^ dm] in cranberry pomace extract powders obtained without carrier addition (**a**), as well as, with different carrier agents obtained by extraction with: 30% acetone (**b**), 50% ethanol (**c**) and acidified 50% ethanol (**d**). FD – freeze-drying; VD60 – vacuum drying at 60 °C; VD90 – vacuum drying at 90 °C; M – maltodextrin; I – inulin; T – trehalose; M-I – blend composed of maltodextrin and inulin; M-T – blend composed of maltodextrin and trehalose; I-T – blend composed of inulin and trehalose; ^a, b, c, …^– different letters within groups: controls, carrier-added samples (acetone), carrier-added samples (50% ethanol), carrier-added samples (50% acidified ethanol) standing for different cranberry pomace extract powders indicate significant differences (ANOVA, HSD Tukey, P < 0.05).

Similarly to White et al. (2010a) and Roopchand et al. (2013), 4 major groups of phenolics were quantified in powders produced without carriers addition, i.e., flavonols (64% of all quantified phenolics), phenolic acids (13.4%), flavan-3-ols (11.6%) and anthocyanins (11%) (Table 2), regardless of the solvent applied and drying technique used. The highest retention of phenolics in controls was noted when acidified 50% ethanol was used, followed by 50% ethanol and 30% acetone, an application of which resulted in, on average, 15% lower content of those constituents in powders (Fig. 1). It was in agreement with Klavins et al. (2022) who recommended acidified ethanol for efficient extraction of phenolics from cranberry press residue. Drying techniques affected phenolics content in the following order: FD < VD60 < VD90. FD and VD60 resulted in products with comparable quantities of all identified phenolics when acidified 50% ethanol was used, while VD90 turned out to be only approx. 8% less effective in terms of phenolics retention. This was in contrast to vacuum drying of cranberry juice extracts that resulted in approx. 30% and 90% lower content of phenolics when temperature of 60 °C and 90 °C was used for powders preparation in comparison to FD (Michalska et al., 2018). This indicates that types of fruit matrix (i.e., juice, pomace) have a significant effect on alterations of phenolics during thermal treatment. Considering their relatively low degradation and a high operating costs of freeze-drying being approx. 50% higher than vacuum drying (Peighambardoust et al., 2011), the latter can be recommended for production of cranberry pomace extracts powders. The application of acidified 50% ethanol (pH = 2) for extracts’ preparation and VD90 led to powders obtained with a higher content of phenolics than in case of products gained by using 30% acetone, even when FD was used. In conclusion, selection of appropriate solvent play a pivotal role in designing of the final quality of cranberry pomace extract powders, and at the same time may enable to reduce costs associated with long-time drying, especially lyophilization (Ratti, 2001).

**Table 2:** Phenolics content [g · 100 g^−1^ dm], antioxidant capacity [mmol Trolox · 100 g^−1^ dm] and hydroxymethyl-*L*-furfural (HMF) content [μg · 100 g^−1^ dm] in cranberry pomace extract control powders.

Solvent type	Drying technique	Phenolics				Antioxidant capacity		Hydroxymethyl-*L*-furfural
		Flavonols	Flavan-3-ols	Phenolic acids	Anthocyanins	TEAC ABTS	FRAP	
30% Acetone	FD	9.62 ± 0.32 ^a-c^	3.01 ± 0.16 ^b^	2.09 ± 0.06 ^ab^	1.66 ± 0.02 ^bc^	398.86 ± 1.35 ^ab^	264.38 ± 2.41 ^a-c^	38.63 ± 1.31 ^b^
	VD60	8.99 ± 0.15 ^a^	2.05 ± 0.00 ^a^	1.94 ± 0.02 ^a^	1.47 ± 0.01 ^ab^	403.28 ± 18.40 ^b^	271.07 ± 12.39 ^a-c^	48.18 ± 3.55 ^c^
	VD90	9.06 ± 0.11 ^ab^	2.34 ± 0.07 ^a^	1.95 ± 0.02 ^a^	1.38 ± 0.05 ^a^	379.98 ± 1.75 ^ab^	259.68 ± 6.44 ^a-c^	47.13 ± 1.56 ^c^
50% Ethanol	FD	12.61 ± 0.06 ^e^	T.A.	2.43 ± 0.01 ^d^	2.28 ± 0.01 ^e^	395.10 ± 18.86 ^ab^	279.84 ± 19.36 ^c^	T.A.
	VD60	11.04 ± 0.39 ^d^	T.A.	2.37 ± 0.09 ^cd^	2.01 ± 0.09 ^d^	375.54 ± 3.37 ^ab^	262.60 ± 9.14 ^a-c^	T.A.
	VD90	10.18 ± 0.44 ^cd^	T.A.	2.03 ± 0.00 ^a^	1.50 ± 0.08 ^ab^	371.77 ± 18.52 ^ab^	273.18 ± 0.41 ^bc^	T.A.
50% Ethanol (pH = 2)	FD	10.87 ± 0.41 ^d^	3.43 ± 0.14 ^c^	2.32 ± 0.06 ^cd^	2.11 ± 0.07 ^de^	363.82 ± 4.70 ^ab^	243.68 ± 0.51 ^ab^	6.80 ± 0.35 ^a^
	VD60	10.84 ± 0.04 ^d^	3.45 ± 0.06 ^c^	2.23 ± 0.00 ^bc^	2.02 ± 0.02 ^d^	358.30 ± 6.17 ^a^	238.69 ± 1.54 ^a^	6.64 ± 0.02 ^a^
	VD90	10.11 ± 0.05 ^b-d^	3.20 ± 0.11 ^bc^	2.09 ± 0.03 ^ab^	1.75 ± 0.03 ^c^	397.69 ± 3.07 ^ab^	276.12 ± 3.18 ^bc^	6.55 ± 0.33 ^a^

FD – freeze-drying; VD60 – vacuum drying at 60 °C; VD90 – vacuum drying at 90 °C; T.A. – trace amount; ^a, b, c, …^– different letters within groups standing for different cranberry pomace extract powders indicate significant differences (ANOVA, HSD Tukey, P < 0.05).

Among four identified groups of phenolics in powders, the highest content of flavonols (approx. 64% of all identified phenolics) was indicated. Extraction solvent influenced their retention that was also moderated by drying technique and parameters. Among solvent applied, 30% acetone resulted in the lowest content of flavonols in powders, regardless of the drying technique used (Fig. 1). Application of 50% ethanol yielded the highest content of these constituents in freeze-dried products followed by samples produced by VD60. In the case of acidified 50% ethanol, no significant differences in powders produced by FD and VD60 were noted, which might indicate flavonols stability during heating (Michalska et al., 2018). Regardless of extraction solvent, difference between an average content of flavonols after FD and VD60 as well as VD90 was, respectively, 8% and 14%.

The third group of phenolics determined in cranberry extracts powders were phenolic acids. In controls, the highest content of these constituents was noted in products gained when 50% ethanol was used in the case of FD and VD60 (Table 2). Application of 30% acetone caused the lowest content of phenolic acids in all powders. FD led to obtainment of powders with the highest content of phenolic acids, whereas the influence of VD differed due to temperature applied for powders production. The temperature of 90 °C caused a lower content of these components in powders for production of which the 50% ethanol and acidified 50% ethanol was used. Interestingly, application of 30% acetone led to powders obtained by VD with similar content of phenolic acids, regardless of the temperature used for powders production.

Previously, cranberry procyanidins belonging to the flavan-3-ols family, were mainly extracted by acetone at different concentrations with acidifying agents used for lowering the pH of an extraction medium (Wallace and Giusti, 2010). It was also proved that absorber technology, including type of resin and solvents used for desorption of components, had an impact on the presence and content of procyanidins in products obtained (Gao et al., 2018), and thus in their powdered form. In the study, for controls, the usage of 50% ethanol resulted in only trace amount of flavan-3-ols in these products, whereas their content in powders obtained with application of 30% acetone and acidified 50% ethanol ranged from 2.05 up to 3.25 mg · 100 g^−1^ dm (Table 2). This confirmed that acidification of the extraction medium significantly improved extractability of cranberry flavan-3-ols. Previously, Klavins et al. (2022) pointed that acidified acetone at various concentrations was the most effective solvent for procyanidins extraction of dried cranberry press residue when compared to ethanolic solution, however, effect of drying and its parameters was neglected. Moreover, the measurement of procyanidins was made by a spectrophotometric method which may not be precise for accurate determination of these constituents. In the study, it was supposed that absorber technology modified composition of initial matrix of cranberry pomace extracts (Gao et al., 2018) which may influence presence of flavan-3-ols in powders gained. Thus, 50% ethanol at pH = 2 can be recommended for production of powders with improved content of flavan-3-ols. It can be concluded that extraction solvent should be chosen on the basis of composition of the material submitted to extraction. In the case of acidified 50% ethanol extracts, drying techniques and parameters used led to obtainment of products with similar content of flavan-3-ols. This indicated a thermal stability of these components extracted with 50% ethanol at pH = 2. One possible explanation might be that different structures of compounds belonging to this group were extracted from cranberry pomace by using specific type of solvent, and therefore with different thermal stability depending on the degree of polymerization (higher polymerization resulted in a lower heat resistance) (Qi et al., 2022).

Among phenolics groups quantified in powders, anthocyanins were present in the smallest quantities (Table 2). Regardless of drying technique, the highest content of these components was noted in products extracted with 50% ethanol and its acidified counterpart (Klavins et al., 2022), that was on average 23% higher when compared to 30% acetone. Drying techniques affected anthocyanins' content as their presence in powders were in the following order: FD > VD60 > VD90. Irrespective of solvent type used, VD60 and VD90 diminished their content, by 9% and 24%, accordingly, compared to FD. Therefore the anthocyanins’ presence, as particularly temperature-sensitive compounds, was noticeably moderated by parameters used during selected drying techniques, which was especially linked to interplay between temperature and time applied (Reque et al., 2016; Zhu et al., 2017).

##### 3.2.2.2 Carrier-added powders

In the next part of the study, powders were produced with addition of carriers in order to verify how the carrier type (maltodextrin, inulin and trehalose) and their binary blends (mixes) affect phenolics stability during drying (Table 3). The addition of carriers at the level of 10% (*w/w*) resulted in, on average, 3.6-times lower content of phenolics (Michalska et al., 2018), in return for higher process efficiency (data not shown). Similarly to controls, a strong influence of solvent type was indicated as acidified 50% ethanol (pH = 2) led to obtainment of powders with a higher content of phenolics that was 28% and 13% higher when compared to those produced using 30% acetone and 50% ethanol as solvents, respectively (Table 3).

**Table 3:** Phenolics content [g · 100 g^−1^ dm], antioxidant capacity [mmol Trolox · 100 g^−1^ dm] and hydroxymethyl-*L*-furfural content [μg · 100 g^−1^ dm] in cranberry pomace extract powders with carriers.

Solvent type	Drying technique	Carrier type	Phenolics				Antioxidant capacity		Hydroxymethyl-*L*-furfural
			Flavonols	Flavan-3-ols	Phenolic acids	Anthocyanins	TEAC ABTS	FRAP	
30% Acetone	FD	M	2.58 ± 0.07 ^d-f^	0.75 ± 0.03 ^cd^	0.56 ± 0.01 ^b^	0.45 ± 0.01 ^ef^	112.48 ± 1.34 ^cd^	78.74 ± 1.10 ^cd^	8.84 ± 0.75 ^a^
		I	2.67 ± 0.01 ^f^	0.82 ± 0.03 ^d^	0.58 ± 0.00 ^b^	0.48 ± 0.00 ^f^	110.65 ± 8.74 ^b-d^	77.92 ± 2.71 ^cd^	10.31 ± 1.02 ^a-c^
		T	2.61 ± 0.09 ^d-f^	0.65 ± 0.00 ^a-d^	0.58 ± 0.02 ^b^	0.47 ± 0.01 ^f^	99.23 ± 6.28 ^a-c^	68.88 ± 4.58 ^a-c^	10.12 ± 1.22 ^a-c^
		M-I	2.57 ± 0.07 ^d-f^	0.67 ± 0.01 ^a-d^	0.58 ± 0.02 ^b^	0.45 ± 0.01 ^ef^	87.96 ± 8.10 ^a^	60.86 ± 6.34 ^a^	16.48 ± 1.04 ^e^
		M-T	2.58 ± 0.12 ^d-f^	0.67 ± 0.04 ^a-d^	0.57 ± 0.02 ^b^	0.46 ± 0.01 ^f^	105.09 ± 0.58 ^b-d^	72.78 ± 0.91 ^b-d^	15.37 ± 0.26 ^de^
		I-T	2.64 ± 0.00 ^ef^	0.68 ± 0.01 ^b-d^	0.58 ± 0.01 ^b^	0.44 ± 0.02 ^ef^	106.16 ± 6.08 ^b-d^	76.09 ± 1.02 ^cd^	16.35 ± 0.01 ^e^
	VD60	M	1.98 ± 0.20 ^a^	0.51 ± 0.02 ^a^	0.47 ± 0.04 ^ab^	0.32 ± 0.04 ^b-d^	108.08 ± 2.29 ^b-d^	72.49 ± 1.39 ^b-d^	10.37 ± 1.70 ^a-c^
		I	2.39 ± 0.09 ^a-f^	0.55 ± 0.02 ^ab^	0.53 ± 0.02 ^ab^	0.39 ± 0.01 ^de^	108.77 ± 0.21 ^b-d^	73.31 ± 1.15 ^b-d^	12.17 ± 0.98 ^a-d^
		T	2.43 ± 0.02 ^b-f^	0.57 ± 0.00 ^ab^	0.55 ± 0.01 ^ab^	0.39 ± 0.01 ^de^	104.08 ± 2.69 ^b-d^	72.87 ± 6.58 ^b-d^	13.19 ± 0.89 ^c-e^
		M-I	2.39 ± 0.18 ^a-f^	0.63 ± 0.03 ^a-c^	0.55 ± 0.04 ^ab^	0.38 ± 0.03 ^c-e^	94.54 ± 1.22 ^ab^	64.09 ± 4.63 ^ab^	11.78 ± 0.64 ^a-d^
		M-T	2.57 ± 0.03 ^d-f^	0.68 ± 0.02 ^a-d^	0.55 ± 0.03 ^ab^	0.41 ± 0.01 ^ef^	110.97 ± 2.15 ^cd^	72.96 ± 0.13 ^b-d^	12.92 ± 0.47 ^b-e^
		I-T	2.43 ± 0.06 ^b-f^	0.64 ± 0.00 ^a-c^	0.56 ± 0.01 ^b^	0.39 ± 0.01 ^de^	106.34 ± 0.02 ^b-d^	75.63 ± 1.24 ^cd^	9.14 ± 0.27 ^a-c^
	VD90	M	2.09 ± 0.03 ^a-c^	0.59 ± 0.05 ^a-c^	0.48 ± 0.02 ^ab^	0.27 ± 0.00 ^b^	106.26 ± 0.92 ^b-d^	73.72 ± 0.11 ^b-d^	9.53 ± 1.09 ^a-c^
		I	2.30 ± 0.14 ^a-f^	0.70 ± 0.07 ^b-d^	0.51 ± 0.08 ^ab^	0.27 ± 0.04 ^b^	109.41 ± 0.62 ^b-d^	78.34 ± 0.18 ^cd^	9.36 ± 1.94 ^a-c^
		T	2.45 ± 0.05 ^c-f^	0.68 ± 0.00 ^a-d^	0.55 ± 0.01 ^ab^	0.27 ± 0.01 ^b^	106.78 ± 5.55 ^b-d^	75.14 ± 1.76 ^b-d^	9.30 ± 0.11 ^a-c^
		M-I	2.21 ± 0.08 ^a-e^	0.62 ± 0.05 ^a-c^	0.51 ± 0.01 ^ab^	0.31 ± 0.02 ^bc^	109.88 ± 1.07 ^b-d^	80.05 ± 1.09 ^cd^	10.50 ± 0.43 ^a-c^
		M-T	2.17 ± 0.08 ^a-d^	0.61 ± 0.01 ^a-c^	0.51 ± 0.02 ^ab^	0.33 ± 0.00 ^b-d^	118.15 ± 3.69 ^d^	82.78 ± 0.02 ^d^	9.05 ± 1.04 ^ab^
		I-T	1.99 ± 0.26 ^ab^	0.56 ± 0.12 ^ab^	0.43 ± 0.06 ^a^	0.18 ± 0.02 ^a^	108.66 ± 1.72 ^b-d^	73.92 ± 1.15 ^b-d^	12.62 ± 1.60 ^a-e^
50% Ethanol	FD	M	3.18 ± 0.04 ^a-e^	T.A.	0.64 ± 0.00 ^a-e^	0.57 ± 0.00 ^e-i^	107.62 ± 1.42 ^ab^	76.79 ± 0.42 ^a-c^	T.A.
		I	3.25 ± 0.11 ^a-f^	T.A.	0.69 ± 0.03 ^a-f^	0.59 ± 0.02 ^f-i^	94.13 ± 5.50 ^a^	68.99 ± 0.72 ^a^	T.A.
		T	3.45 ± 0.20 ^c-f^	T.A.	0.71 ± 0.03 ^c-f^	0.60 ± 0.03 ^g-i^	112.07 ± 2.39 ^b^	79.72 ± 0.65 ^a-c^	T.A.
		M-I	3.45 ± 0.07 ^c-f^	T.A.	0.72 ± 0.02 ^c-f^	0.62 ± 0.02 ^hi^	108.30 ± 2.00 ^ab^	72.40 ± 1.61 ^a-c^	T.A.
		M-T	3.68 ± 0.01 ^f^	T.A.	0.77 ± 0.01 ^f^	0.66 ± 0.01 ^i^	110.32 ± 2.34 ^b^	79.99 ± 0.77 ^bc^	T.A.
		I-T	3.54 ± 0.03 ^d-f^	T.A.	0.73 ± 0.00 ^d-f^	0.64 ± 0.01 ^hi^	108.98 ± 0.14 ^ab^	77.46 ± 2.64 ^a-c^	T.A.
	VD60	M	3.09 ± 0.03 ^a-d^	T.A.	0.67 ± 0.00 ^a-e^	0.51 ± 0.02 ^d-f^	99.70 ± 12.27 ^ab^	71.48 ± 7.18 ^ab^	T.A.
		I	2.99 ± 0.03 ^a-c^	T.A.	0.64 ± 0.00 ^a-d^	0.52 ± 0.01 ^d-g^	104.43 ± 4.26 ^ab^	73.97 ± 3.93 ^a-c^	T.A.
		T	3.24 ± 0.02 ^a-f^	T.A.	0.69 ± 0.00 ^b-f^	0.56 ± 0.00 ^e-h^	109.70 ± 2.49 ^b^	78.73 ± 0.42 ^a-c^	T.A.
		M-I	3.02 ± 0.01 ^a-c^	T.A.	0.64 ± 0.00 ^a-c^	0.53 ± 0.00 ^d-g^	107.02 ± 1.03 ^ab^	77.18 ± 5.12 ^a-c^	T.A.
		M-T	3.58 ± 0.04 ^ef^	T.A.	0.74 ± 0.01 ^ef^	0.61 ± 0.02 ^hi^	107.76 ± 1.48 ^ab^	77.38 ± 1.25 ^a-c^	T.A.
		I-T	3.34 ± 0.04 ^b-f^	T.A.	0.70 ± 0.00 ^b-f^	0.55 ± 0.01 ^e-h^	108.06 ± 0.15 ^ab^	78.17 ± 1.62 ^a-c^	T.A.
	VD90	M	2.93 ± 0.24 ^ab^	T.A.	0.65 ± 0.07 ^a-e^	0.48 ± 0.06 ^c-e^	103.86 ± 1.63 ^ab^	73.41 ± 1.95 ^a-c^	T.A.
		I	3.07 ± 0.01 ^a-d^	T.A.	0.65 ± 0.00 ^a-e^	0.46 ± 0.01 ^b-d^	108.56 ± 0.94 ^ab^	78.06 ± 1.79 ^a-c^	T.A.
		T	3.44 ± 0.00 ^c-f^	T.A.	0.71 ± 0.00 ^c-f^	0.43 ± 0.01 ^bc^	112.27 ± 0.39 ^b^	78.39 ± 0.90 ^a-c^	T.A.
		M-I	2.82 ± 0.12 ^a^	T.A.	0.60 ± 0.01 ^ab^	0.39 ± 0.01 ^b^	107.90 ± 4.13 ^ab^	75.08 ± 3.71 ^a-c^	T.A.
		M-T	3.24 ± 0.33 ^a-f^	T.A.	0.68 ± 0.06 ^a-f^	0.51 ± 0.04 ^c-f^	114.10 ± 2.00 ^b^	82.86 ± 1.63 ^c^	T.A.
		I-T	3.11 ± 0.01 ^a-e^	T.A.	0.60 ± 0.00 ^a^	0.24 ± 0.00 ^a^	112.53 ± 0.32 ^b^	78.37 ± 0.65 ^a-c^	T.A.
50% Ethanol (pH = 2)	FD	M	3.23 ± 0.00 ^e-g^	1.04 ± 0.00 ^e-g^	0.67 ± 0.00 ^f-h^	0.61 ± 0.01 ^g-i^	104.32 ± 2.36 ^a-e^	71.83 ± 3.38 ^a-d^	3.96 ± 0.16 ^a-c^
		I	2.89 ± 0.05 ^a-e^	0.93 ± 0.00 ^b-f^	0.59 ± 0.01 ^b-e^	0.55 ± 0.01 ^e-g^	97.18 ± 0.37 ^ab^	68.60 ± 0.37 ^ab^	3.68 ± 0.03 ^a-c^
		T	3.37 ± 0.07 ^fg^	1.06 ± 0.04 ^fg^	0.68 ± 0.01 ^gh^	0.64 ± 0.01 ^hi^	112.75 ± 0.91 ^c-f^	79.30 ± 0.98 ^cd^	4.09 ± 0.48 ^c^
		M-I	3.44 ± 0.02 ^g^	1.07 ± 0.00 ^g^	0.71 ± 0.00 ^h^	0.66 ± 0.00 ^i^	111.58 ± 1.55 ^c-f^	78.68 ± 1.42 ^b-d^	4.29 ± 0.48 ^c^
		M-T	3.23 ± 0.04 ^e-g^	1.01 ± 0.02 ^d-g^	0.66 ± 0.01 ^e-h^	0.61 ± 0.01 ^f-i^	104.24 ± 2.03 ^a-e^	68.84 ± 2.44 ^ab^	4.04 ± 0.08 ^bc^
		I-T	2.99 ± 0.02 ^b-g^	0.95 ± 0.02 ^c-g^	0.62 ± 0.00 ^b-g^	0.57 ± 0.01 ^e-h^	105.62 ± 0.57 ^a-e^	70.79 ± 0.62 ^a-c^	3.58 ± 0.21 ^a-c^
	VD60	M	2.64 ± 0.02 ^a-c^	0.85 ± 0.00 ^bc^	0.57 ± 0.00 ^a-c^	0.45 ± 0.01 ^bc^	105.63 ± 1.25 ^a-e^	76.50 ± 2.34 ^a-d^	3.89 ± 0.17 ^a-c^
		I	2.95 ± 0.02 ^a-f^	0.98 ± 0.02 ^c-g^	0.63 ± 0.00 ^b-g^	0.54 ± 0.00 ^d-f^	103.68 ± 1.36 ^a-d^	67.51 ± 5.52 ^a^	3.67 ± 0.02 ^a-c^
		T	3.14 ± 0.02 ^d-g^	1.00 ± 0.01 ^d-g^	0.67 ± 0.01 ^e-h^	0.58 ± 0.01 ^e-h^	111.30 ± 1.07 ^c-f^	78.36 ± 0.11 ^b-d^	4.21 ± 0.10 ^c^
		M-I	2.90 ± 0.06 ^a-e^	0.94 ± 0.01 ^b-g^	0.62 ± 0.01 ^b-g^	0.54 ± 0.01 ^d-f^	108.29 ± 6.52 ^b-f^	75.69 ± 4.03 ^a-d^	3.82 ± 0.02 ^a-c^
		M-T	3.05 ± 0.19 ^b-g^	0.96 ± 0.04 ^c-g^	0.65 ± 0.03 ^c-h^	0.54 ± 0.03 ^d-g^	111.24 ± 0.66 ^c-f^	73.60 ± 5.27 ^a-d^	3.72 ± 0.30 ^a-c^
		I-T	2.69 ± 0.05 ^a-d^	0.87 ± 0.00 ^bc^	0.58 ± 0.00 ^b-d^	0.48 ± 0.01 ^cd^	101.75 ± 1.89 ^a-c^	68.53 ± 1.09 ^ab^	3.77 ± 0.30 ^a-c^
	VD90	M	3.08 ± 0.15 ^c-g^	0.95 ± 0.05 ^c-g^	0.65 ± 0.02 ^d-h^	0.51 ± 0.03 ^c-e^	118.65 ± 3.36 ^f^	81.94 ± 0.66 ^d^	3.87 ± 0.05 ^a-c^
		I	2.59 ± 0.18 ^ab^	0.81 ± 0.05 ^ab^	0.55 ± 0.04 ^ab^	0.39 ± 0.03 ^b^	96.84 ± 6.17 ^a^	70.79 ± 2.63 ^a-c^	3.75 ± 0.63 ^a-c^
		T	3.10 ± 0.20 ^c-g^	0.91 ± 0.05 ^b-e^	0.65 ± 0.03 ^d-h^	0.45 ± 0.02 ^bc^	113.62 ± 5.10 ^d-f^	79.54 ± 1.04 ^cd^	2.93 ± 0.07 ^a^
		M-I	2.77 ± 0.16 ^a-e^	0.91 ± 0.04 ^b-e^	0.60 ± 0.03 ^b-f^	0.48 ± 0.02 ^cd^	115.03 ± 0.99 ^ef^	78.79 ± 1.66 ^b-d^	3.38 ± 0.04 ^a-c^
		M-T	2.81 ± 0.24 ^a-e^	0.88 ± 0.08 ^b-d^	0.60 ± 0.03 ^b-f^	0.48 ± 0.03 ^cd^	109.66 ± 0.21 ^c-f^	77.99 ± 1.44 ^b-d^	2.98 ± 0.03 ^ab^
		I-T	2.51 ± 0.11 ^a^	0.70 ± 0.03 ^a^	0.50 ± 0.02 ^a^	0.20 ± 0.02 ^a^	109.16 ± 0.32 ^c-f^	77.69 ± 2.12 ^a-d^	3.92 ± 0.33 ^a-c^

FD – freeze-drying; VD60 – vacuum drying at 60 °C; VD90 – vacuum drying at 90 °C; M – maltodextrin; I – inulin; T – trehalose; M-I – blend composed of maltodextrin and inulin; M-T – blend composed of maltodextrin and trehalose; I-T – blend composed of inulin and trehalose; T.A. – trace amount; ^a, b, c, …^– different letters within groups: carrier-added samples (30% acetone), carrier-added samples (50% ethanol), carrier-added samples (50% acidified ethanol) standing for different cranberry pomace extract powders indicate significant differences (ANOVA, HSD Tukey, P < 0.05).

In general, content of all identified phenolics was more affected by drying technique than the carrier type used (one-dimensional significance test; data not shown). FD led to powders with the highest content of phenolics, followed by VD60 and VD90 (decrease of, respectively, 10% and 17%). That was more linked to the content of flavonols and flavan-3-ols, while considerably greater differences were noted for anthocyanins - in this case the degradation was at the level of 15% (VD60) and 51% (VD90) compared to freeze-dried powders. Among single carriers applied, trehalose resulted in the highest retention of sum of identified phenolics in powders gained, regardless of solvent used and drying technique applied. Among carrier blends used for powders production, the I-T binary blend led to lower content of phenolics than M-T, regardless of extraction solvent and drying technique. This composition of blend (M-T) also resulted in the highest content of total phenolics in chokeberry pomace extracts powders (Michalska-Ciechanowska et al., 2021a).

Going into the details, addition of carriers and their blends influenced the amount of compounds that belong to different phenolic’ groups. In the case of flavonols, such an approach led to their lower content, similarly to total phenolics content, approx. 3.6-times the content of flavonols in powders. In general, the sum of flavonols was the highest in powders produced using pomace extracted with 50% ethanol, that was on average 9.4% and 36% higher than in powders produced with acidified 50% ethanol and 30% acetone, respectively, regardless carrier type. Among single carriers, trehalose preserved the flavonols to the highest extent when compared to maltodextrin and inulin. Similarly to the sum of phenolics, addition of a M-T blend resulted in a higher content of flavonols in powders when compared to I-T. When drying technique was concerned, the highest content of flavonols was noted after FD, regardless of the carrier type used, that was higher of approx. 9% and 13% when compared to VD60 and VD90, respectively. Difference between flavonols content during VD60 and VD90 was less than 5%, which was similar to the difference between powders produced without a carrier. This indicated that the carriers did not play a decisive role in flavonols protection during thermal processes.

For flavan-3-ols, when the carrier-added powders were concerned, the solvent type played the most influential role as powders prepared from acidified 50% ethanol-derived extracts had approx. 30% more flavan-3-ols than those obtained with 30% acetone application. As with the control, again extraction with only 50% ethanol yielded residual amounts of flavan-3-ols identified in carrier-added powders, compared to 30% acetone and 50% acidified ethanol. FD gave products with the greatest amount of these constituents, while VD60 and VD90 resulted in their comparable content, which were about 12% lower than lyophilized samples. Amongst applied carriers and its mixes, i.a., trehalose, maltodextrin and their blend seemed to ensure the highest protection during drying, regardless of technique or solvent type. Previously, trehalose was indicated to stabilize selected phenolics, e.g., anthocyanins by ensuring thermal protection during elevated-temperature treatment by forming hydrogen bonds with them and therefore hindering the structure breakdown (Castagnini et al., 2021). Moreover, this substance was indicated as distinctive amongst others due to its superior ability to replace water molecules during dehydration process *via* hydrogen-bonding to biomolecules (Lerbret et al., 2005) known as a ‘water replacement’ hypothesis proposed by Crowe et al. (1994).

Similarly to controls, solvent type had the strongest influence on extraction of phenolic acids. Application of 30% acetone resulted in the lowest content of these compounds, regardless of the type of carrier used for drying, while 50% ethanol and its acidified counterpart gave products with, on average, 27% and 16% higher content, respectively. The drying technique had a slight effect on phenolic acid sum, since compared to freeze-drying, VD60 and VD90 only resulted in a 6% and 11% reduction in their content. Previously, Szwajgier et al. (2014) pointed out that depending on fruit type and its processing, the content of phenolic acids could decrease, as well as remain unchanged or even increase. In addition, composition of matrix in which the individual phenolic acids occur was singled out as one of the key factors. Recognizing the impact of carrier type, the most preferable levels of phenolic acids were found when trehalose and M-T mixture were used for powders production. However, taking into account powders obtained by a given drying technique, freeze-dried products were characterized by a comparable content of phenolic acids (7% difference between the lowest and highest content depending on the carrier used), while in the case of VD60 and VD90, the difference was 14% and 25%, respectively.

In comparison to controls, the average sum of anthocyanins in powders produced with 10% carrier addition was 3.8-times lower. Compared to lyophilizates, products after VD60 and VD90 retained 16 and 34% less of these constituents, respectively, despite the inclusion of protective substances. Intriguingly, despite the contrasting levels of these compounds in control powders and those with a carrier, the percentage difference in the decrease in anthocyanin content caused by high drying temperature, is markedly smaller in products where no protective substance - a carrier - was used (9% and 24% lower content, respectively, for VD60 and VD90 samples compared to freeze-dried ones). Such phenomenon may be linked with other protective mechanisms which could take place in such a phenolic-dense matrix under thermal processing, as copigmentation of anthocyanins by other compounds (Gençdağ et al., 2022). In general, among single carriers, maltodextrin addition resulted in lowest content of anthocyanins when compared to trehalose which led to powders with the highest sum of these constituents (Castagnini et al., 2021). Among diblends the average content of anthocyanins was the lowest when I-T mix was applied, while M-T blend turned out to ensure the greatest protection for this group.

#### 3.2.3 Antioxidant capacity

The antioxidant capacity of extract powders ranged between 88 and 403 and between 61 and 280 mmol Trolox · 100 g^−1^ dm for TEAC ABTS and FRAP measurements, respectively (Table 2, Table 3). Despite the discrepancies in results mainly reflected in lower antioxidant capacity values measured by FRAP method compared to TEAC ABTS assay, which can be attributed to the different reaction mechanisms underlying the principle of the employed methods (Pérez-Burillo et al., 2019), a very similar pattern was observed among the samples.

##### 3.2.3.1 Controls

For control powders, acidified 50% ethanol extraction resulted in the lowest average values of antioxidant capacity measured by both assays, while the highest were noted for those obtained with acetone pomace extraction in case of TEAC ABTS measurement and by 50% ethanol for FRAP method, regardless of drying technique. Independently of solvent type, the VD60 led to powders with the lowest antioxidant properties, while interestingly, lyophilization and VD90 yielded products with relatively higher average values. However, it needs to be noted that differences between discussed results are not substantive, and all control samples exerted antioxidant potential on a comparable level. In general, control powders exerted the highest values, while carrier addition caused approximately 70% lower antioxidant capacity. One reason for this is the lower ratio of extract in the drying solution (90%; *w/w*) compared to the control samples where the extract was 100% (*w/w*). Another possible explanation is the high ability of encapsulating materials to protect bioactives (strong phenolics-wall material interaction) resulting in a lower response in the *in vitro* tests, compared to unencapsulated samples (Radünz et al., 2019).

##### 3.2.3.2 Carrier-added powders

For carrier-added products, solvent type did not affect the antioxidative effect exhibited by these powders to the same extent as in case of phenolics content or HMF formation (described below), and therefore differences between recorded average values were barely noticeable. Taking into account drying technique, the strongest antioxidant capacity was observed for VD90, while FD and VD60 gave comparable quality powders, regardless of solvent type. This in turn may be connected to formation of other Maillard reaction and/or caramelization products such as melanoidins which exhibit varying antioxidant potential (Feumba Dibanda et al., 2020). Despite emerging statistically significant differences between the powders with carrier addition, there are no clear distinctions or recurring patterns depending on the specific type of carrier/binary mixture. However, taking into account TEAC ABTS results, it is worth noting that all samples exhibited considerably higher antioxidant potential compared to cranberry juice powders with 15% maltodextrin addition (1.21–5.15 mmol Trolox · 100 g^−1^ dm) or sugar-free juice extract powders without carrier agent (71.86–188.83 mmol Trolox · 100 g^−1^ dm) obtained by analogous drying methods (Michalska et al., 2018). Therefore, it can be concluded that sustainable pomace extract powders obtained both, with and without addition of an encapsulating substance, constitute a high-value additive with potentially functional properties, which may be used for improving the quality of the foodstuff.

#### 3.2.4 HMF determination

The presence of hydroxymethyl-*L*-furfural was confirmed in powders gained after extraction of cranberry pomace in 30% acetone and acidified 50% ethanol, regardless of drying technique. In carrier-free powders, the HMF content was 6.7-times higher in 30% acetone extracts’ products when compared to those produced after usage of acidified 50% ethanol (Table 2). This was in line with the finding of Bicker et al. (2003) who indicated that water and acetone mixture promote the rearrangement of carbohydrates to furanoid form influencing formation of HMF. Only a trace amount of HMF was detected in 50% ethanolic extract powders. Thus, the solvent applied for cranberry pomace extraction played a key role in moderation of chemical composition of powders produced. Drying had an influence on HMF content in powders. Similarly to Michalska-Ciechanowska et al. (2021a) the formation of HMF was confirmed in freeze-dried fruit-based products. Herein, the usage of 30% acetone for powders production by FD resulted in products with 23.4% lower content of this process contaminant than VD, regardless of temperature applied. The difference between VD60 and VD90 was not statistically significant. In the case of acidified 50% ethanol extracts, drying technique did not differentiate powders in terms of HMF content**.** It can therefore be speculated that due to increased stability of anthocyanins caused by acidic environment during their extraction from the pomace, there was no such significant deglycosylation at later stages, including drying, resulting in the detachment of sugar residues, which might constitute substrate for HMF formation (Ertan et al., 2019).

The average HMF content in products gained with carriers was approx. 4-times and 2-times lower in, respectively, powders obtained after 30% acetone and acidified 50% ethanol cranberry pomace extraction (Table 3). Thus, carrier application diminished the HMF formation in products gained. Among carriers used, the highest content of this compound was noted for powders with M-I blend while application of maltodextrin exclusively resulted in the lowest average HMF content, regardless of the extraction solvent or drying method. In terms of drying technique, the highest average HMF content was found for freeze-dried powders, while VD60 and VD90 reduced it by about 8% and 20%, respectively. This phenomenon may be attributed to longer time of dehydration during lyophilization (24 h) compared to VD60 (22 h) and VD90 (16 h), despite the temperature difference (Fitzpatrick et al., 2013).

### 3.3 Chemometric analysis

**Fig. 2:**
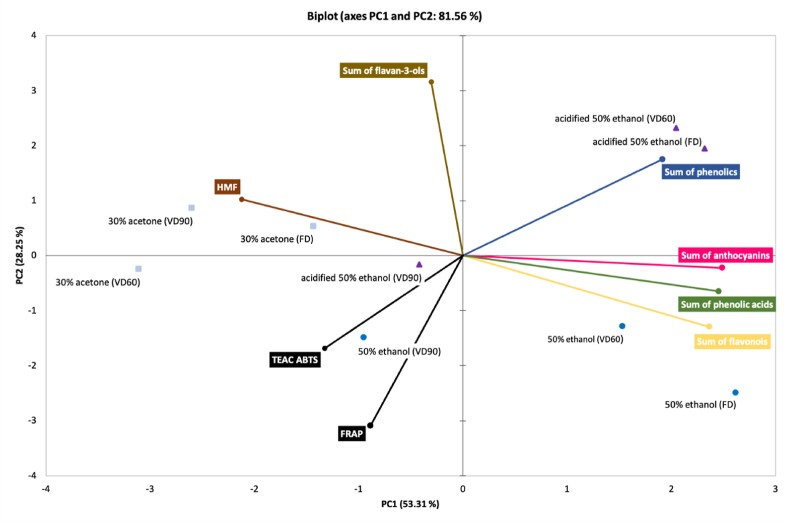
Principal Components Analysis (PCA) biplot that indicates principal components (PC) scores of cranberry extracts (– 30% acetone,  – 50% ethanol,  – acidified 50% ethanol) powders gained by freeze- and vacuum drying. FD – freeze-drying, VD60 – vacuum drying at 60 °C, VD90 – vacuum drying at 90 °C.

The Principal Component Analysis (PCA) was carried out to distinguish relation between phenolics content, including the flavan-3-ols, flavonols, phenolic acids anthocyanins, antioxidant properties (TEAC ABTS and FRAP) and the content of HMF in powders considering different solvent used for cranberry pomace extraction and drying techniques. For control samples (no carrier addition), two principal components (PC) that explained 81.56% of the total variance were chosen (Fig. 2). The PCA biplot showed that control powders can be grouped into: (1) products made with application of acidified 50% ethanol and dried by FD and VD60, (2) powders produced from 50% ethanolic extracts and dried by FD and VD60 and (3) the rest of powders gained. It can be concluded that powders belong to the first group are similar in the content of identified phenolics, whereas the second group can be characterized in similar content of flavonols and phenolic acids. Application of VD90, in the case of 50% ethanol and its acidified counterpart, influence the powders properties that was closed to those produced with acetone.

**Fig. 3:**
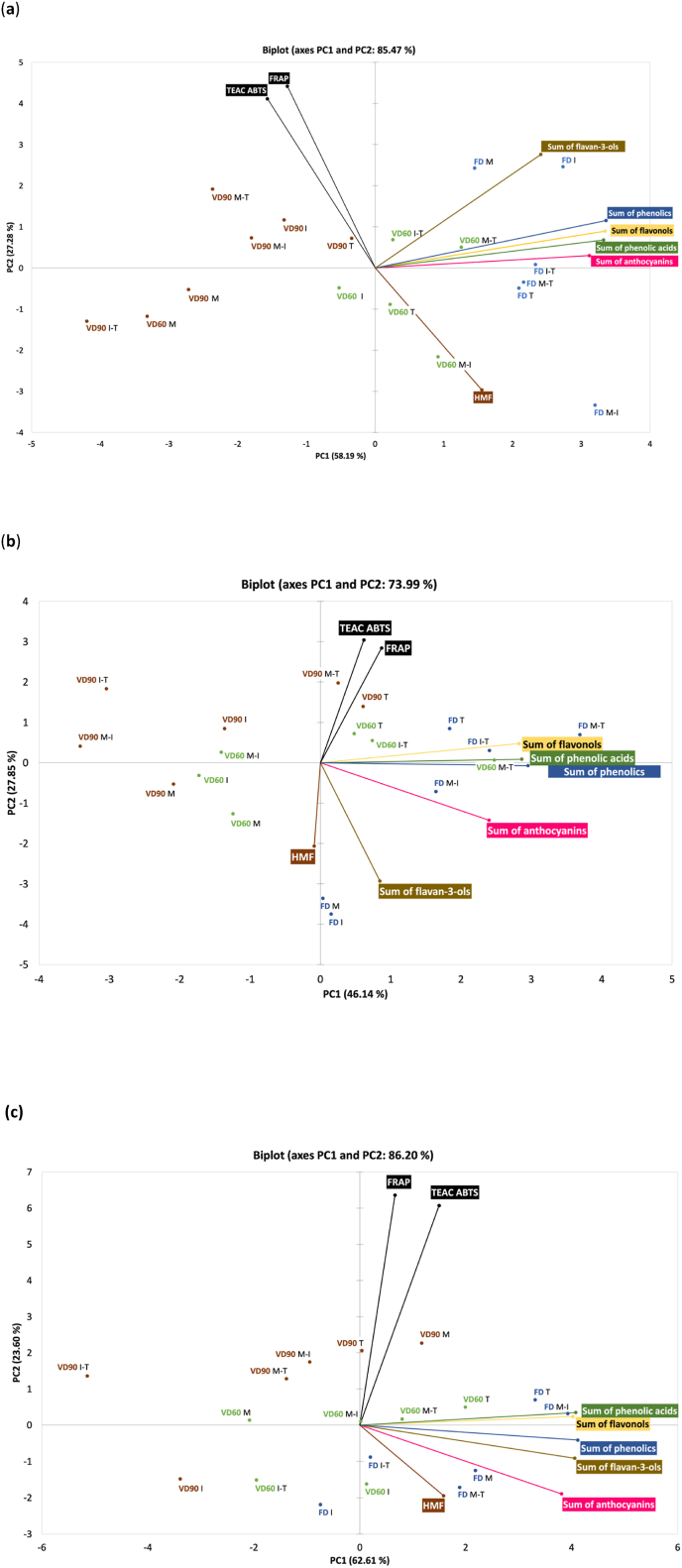
Principal Components Analysis (PCA) biplot that shows principal components (PC) scores of cranberry (**a**) 30% acetone, (**b**) 50% ethanol, and (**c**) 50% acidified ethanol extracts powders gained after freeze- and vacuum drying with addition of carriers. FD – freeze-drying, VD60 – vacuum drying at 60 °C, VD90 – vacuum drying at 90 °C; M – maltodextrin, I – inulin, T – trehalose, M-I – blend composed of maltodextrin and inulin, M-T – blend composed of maltodextrin and trehalose, I-T – blend composed of inulin and trehalose.

Fig. 3a, b and 3c showed how the type of carrier and drying techniques differentiate the cranberry pomace extracts powders. In the case of powders produced from 30% acetone extract (Fig. 3a), the drying techniques significantly moderated the composition of powders pointing that products gained after FD and VD60, except sample obtained after VD60 with inulin addition, were more similar to each other than powders obtained after VD90. This confirmed that when the addition of carriers is considered, the powders can be similar in terms of phenolics after FD and VD60 that is linked to the type of carrier used for drying.

In the case of 50% ethanol cranberry pomace extracts (Fig. 3b), drying techniques did not differentiate powders so clearly as in the case of acetone. Samples gained after FD, except those produced with addition of maltodextrin and inulin, were more similar in terms of phenolics and antioxidant capacity to powders gained after VD60 with trehalose addition as single carrier and its blend with inulin and maltodextrin and to products obtained after VD90 with trehalose and M-T blend. VD60 and VD90 produced powders with similar properties when inulin and maltodextrin (or their blends) were used.

Acidification of 50% ethanol moderated powders properties that was additionally affected by drying techniques and carrier type (Fig. 3c). Changes in chemical composition caused by solvent type resulted in differences between powders quality mainly reflected in procyanidins and HMF content compared to non-acidified counterpart. VD90 resulted in the lowest retention of groups of phenolics, but at the same time the lowest content of HMF, while being the highest in samples after FD with maltodextrin and its blend with trehalose.

## 4 Conclusions

The feasibility of managing cranberry pomace into high-quality soluble powders that might serve as potentially functional food additives, was confirmed. The vacuum-dried products had the most desired physical parameters, while freeze-drying yielded powders with the highest *Mc* and *a*_*w*_, and at the same time the lowest bulk density. Acidified 50% ethanol was proven to be the most effective extracting solvent for cranberry pomace resulting in the highest content of phenolics in powders, while 30% acetone in the lowest amount, and significantly higher hydroxymethyl-*L*-furfural content (up to 48.18 μg · 100 g^−1^ dm). Non-acidified 50% ethanol yielded powders without HMF presence, however along with only trace amounts of flavan-3-ols. The carrier application diminished HMF formation even down to 74%, but at the same time reduced antioxidant capacity equally, with regard to controls. Amongst carriers tested, the highest sum of phenolics was recorded for trehalose (among single carriers) and maltodextrin-trehalose (among blends). It can be suggested that with appropriate optimization strategies, the proposed approach has promising prospects for application to management of other types of waste from fruit and vegetable industry.

## CRediT authorship contribution statement

**Jessica Brzezowska:** Conceptualization, Funding acquisition, Project administration, Methodology, Investigation, Writing – original draft, Writing – review & editing. **Aleksandra Hendrysiak:** Investigation. **Aneta Wojdyło:** Methodology, Investigation, Writing – review & editing. **Anna Michalska-Ciechanowska:** Conceptualization, Supervision, Methodology, Investigation, Writing – original draft, Writing – review & editing.

## Declaration of competing interest

The authors declare that they have no known competing financial interests or personal relationships that could have appeared to influence the work reported in this paper.

## Data Availability

Data will be made available on request.
